# Supplementary figures and images for: Structures of RecBCD in complex with phage-encoded inhibitor proteins reveal distinctive strategies for evasion of a bacterial immunity hub
Source: eLife. 2022 Dec 19;11:e83409. doi: 10.7554/eLife.83409 (PMC9836394; doi:10.7554/eLife.83409)

From Figure 1A

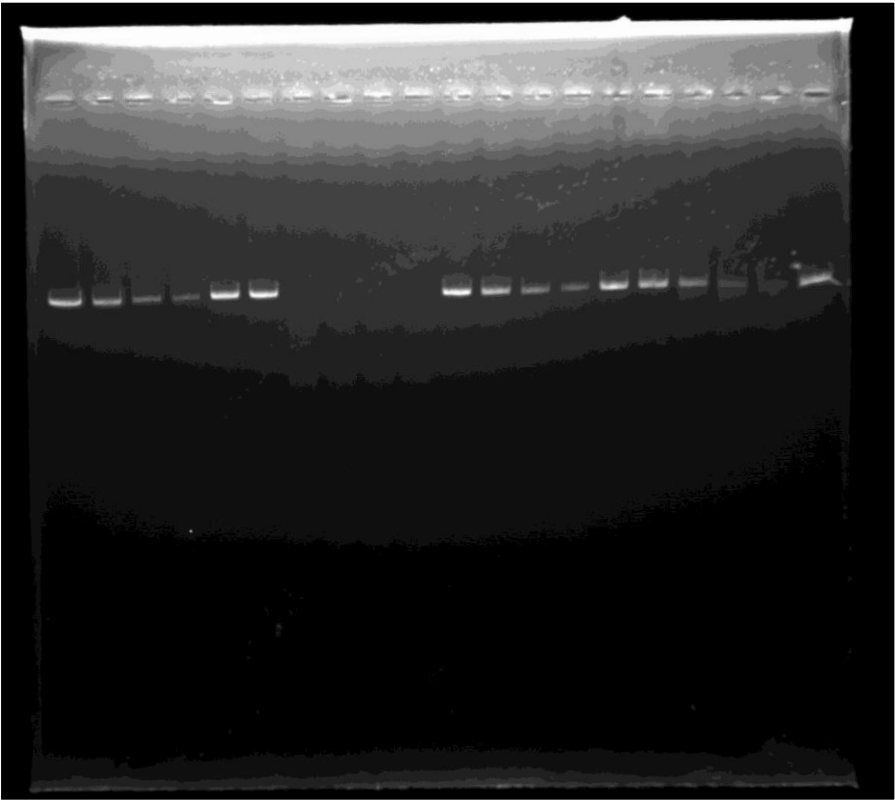

Linearised plasmid  
DNA substrate

From Figure 1E

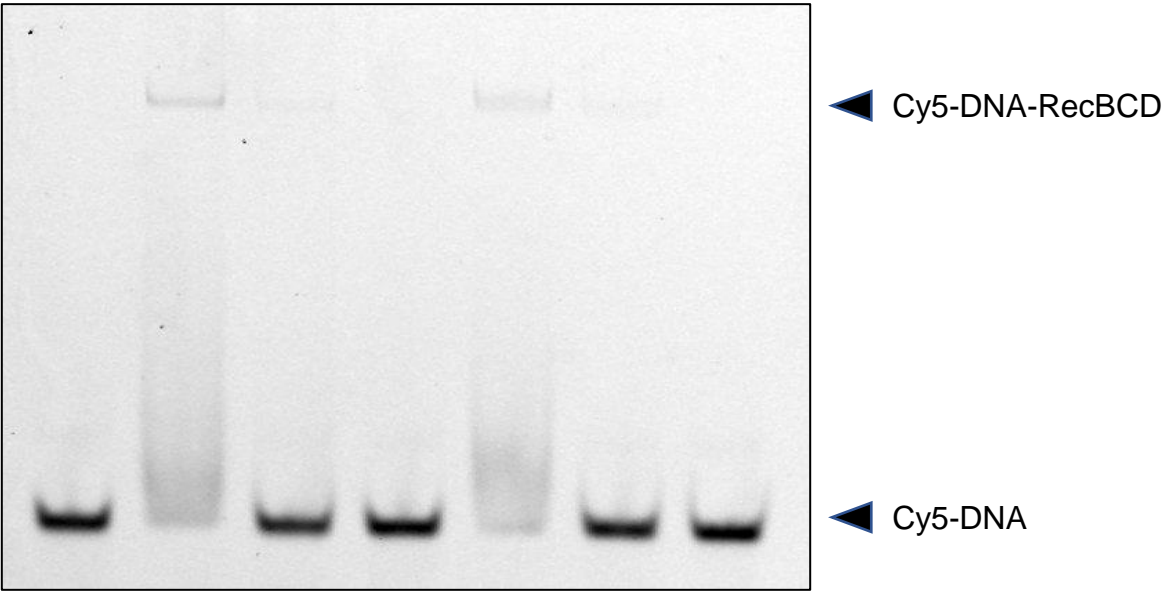

From Figure 1F

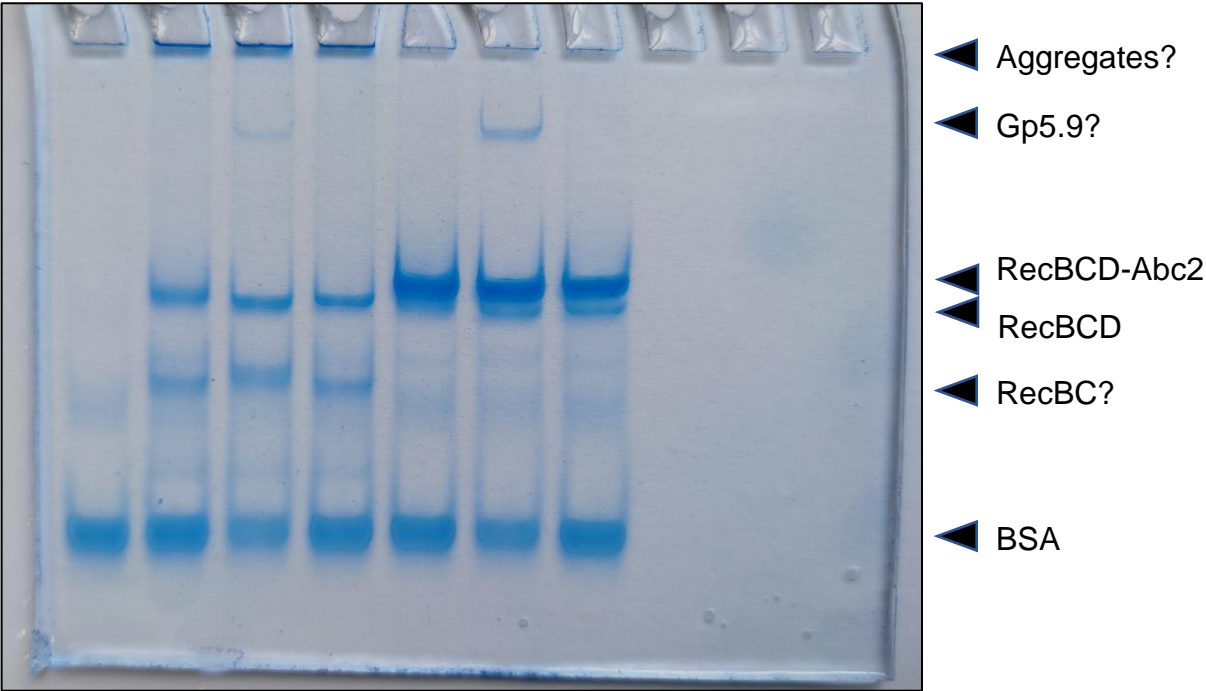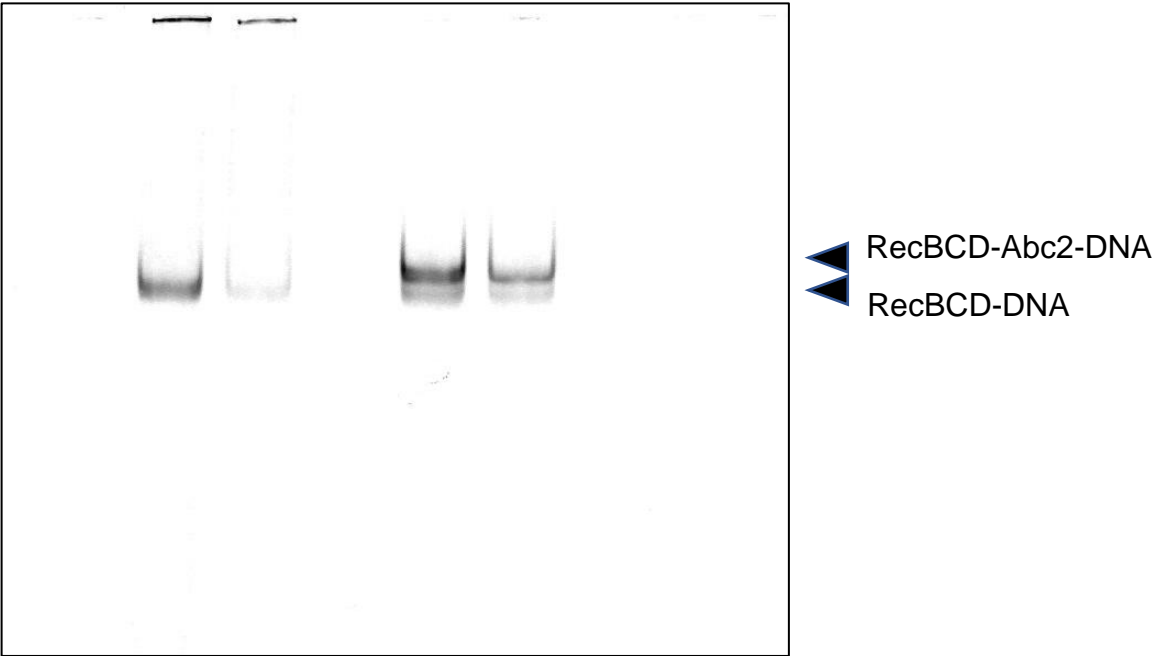

Supplement: Figure 1—source data 1. [file elife-83409-fig1-data1.zip › FIgure 1 - Source Data/All raw gel images with labelled bands.pdf]

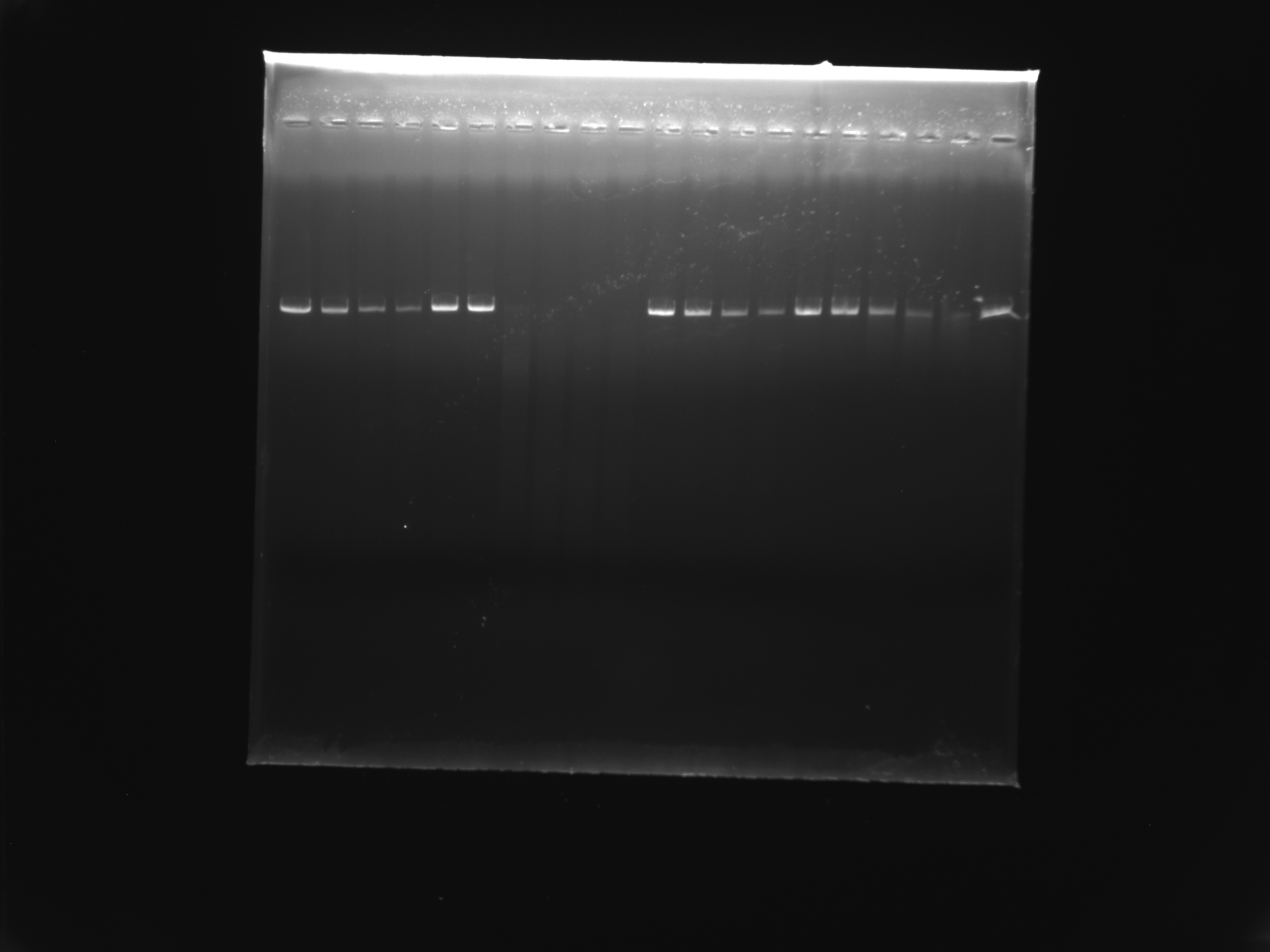

Supplement: Figure 1—source data 1. [file elife-83409-fig1-data1.zip › FIgure 1 - Source Data/RecBCD assay full gel 080322_Figure1A.TIF]

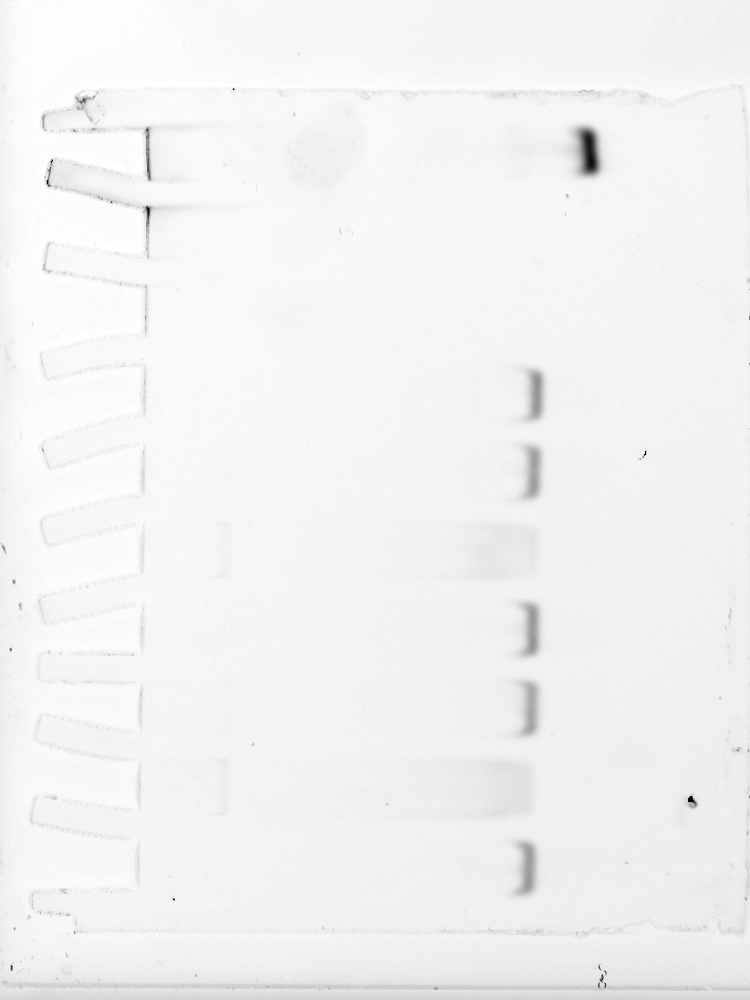

Supplement: Figure 1—source data 1. [file elife-83409-fig1-data1.zip › FIgure 1 - Source Data/RecBCD EMSA 140622-[Cy5] Figure 1E.gel]

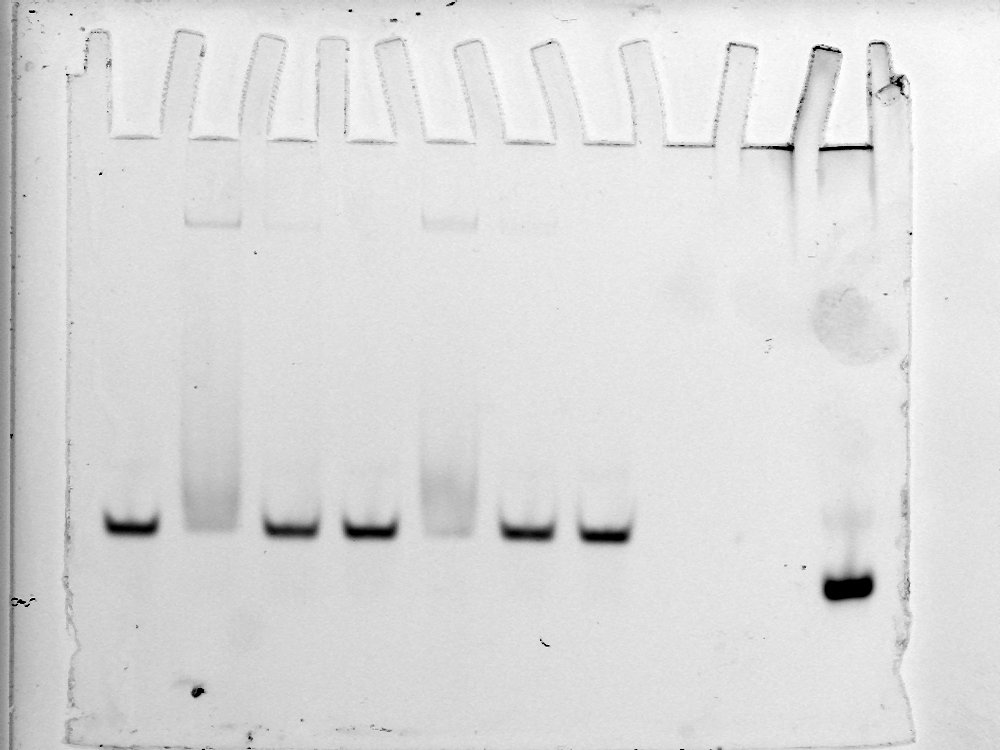

Supplement: Figure 1—source data 1. [file elife-83409-fig1-data1.zip › FIgure 1 - Source Data/RecBCD EMSA 140622-[Cy5] Figure 1E.jpg]

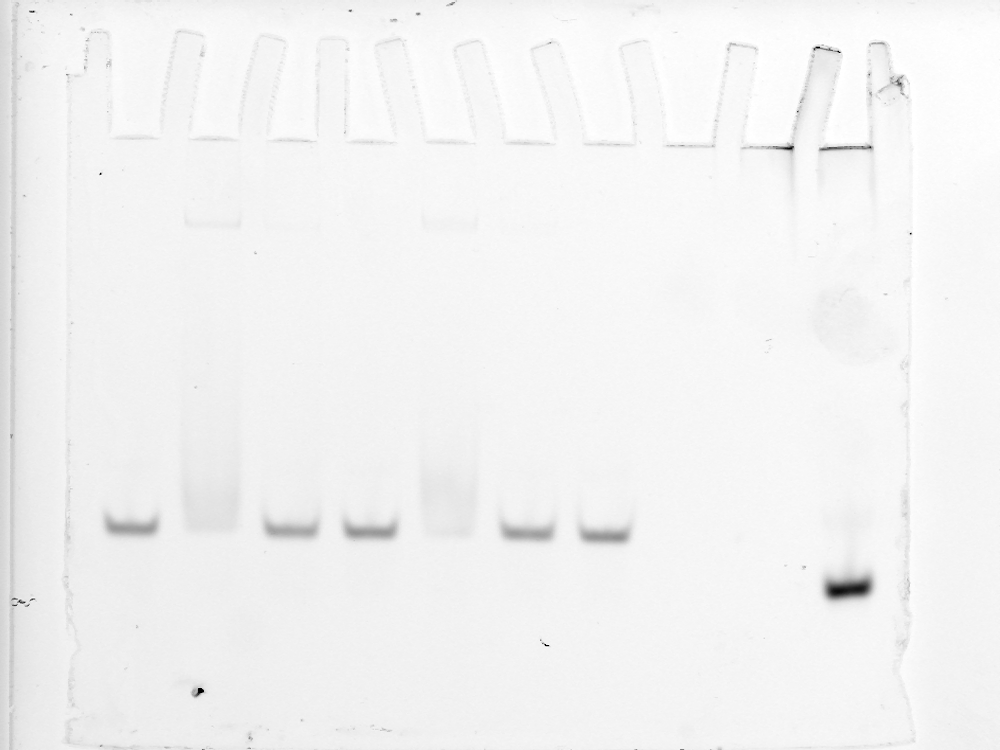

Supplement: Figure 1—source data 1. [file elife-83409-fig1-data1.zip › FIgure 1 - Source Data/RecBCD EMSA 140622-[Cy5] Figure 1E.tif]

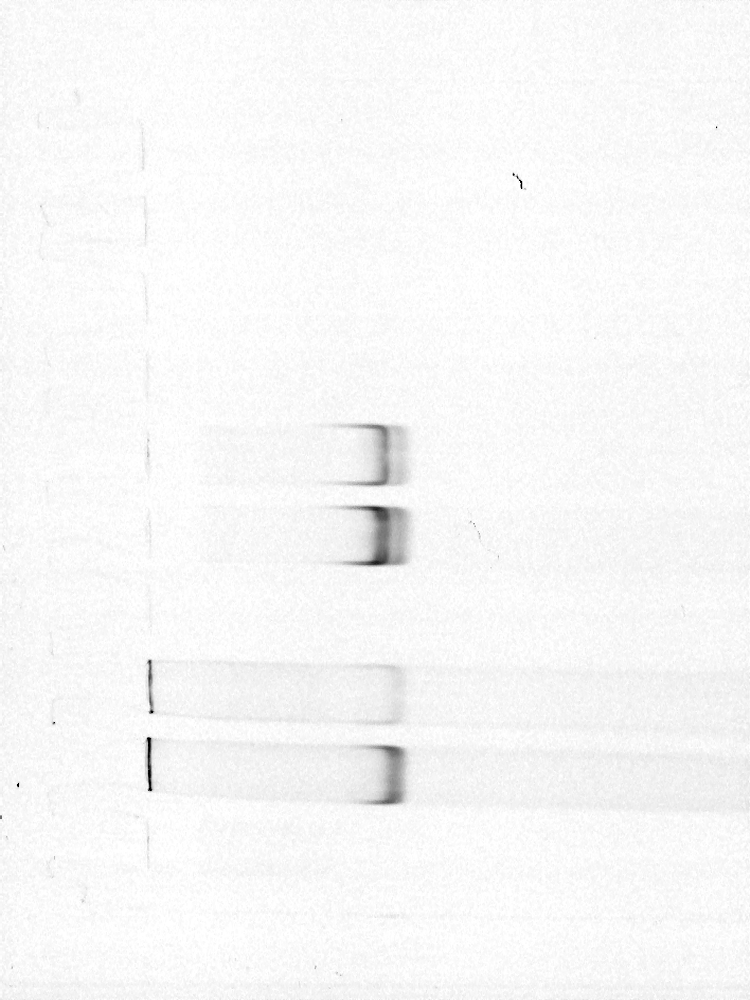

Supplement: Figure 1—source data 1. [file elife-83409-fig1-data1.zip › FIgure 1 - Source Data/RecBCD EMSA 170622-[Cy5] Figure1F.gel]

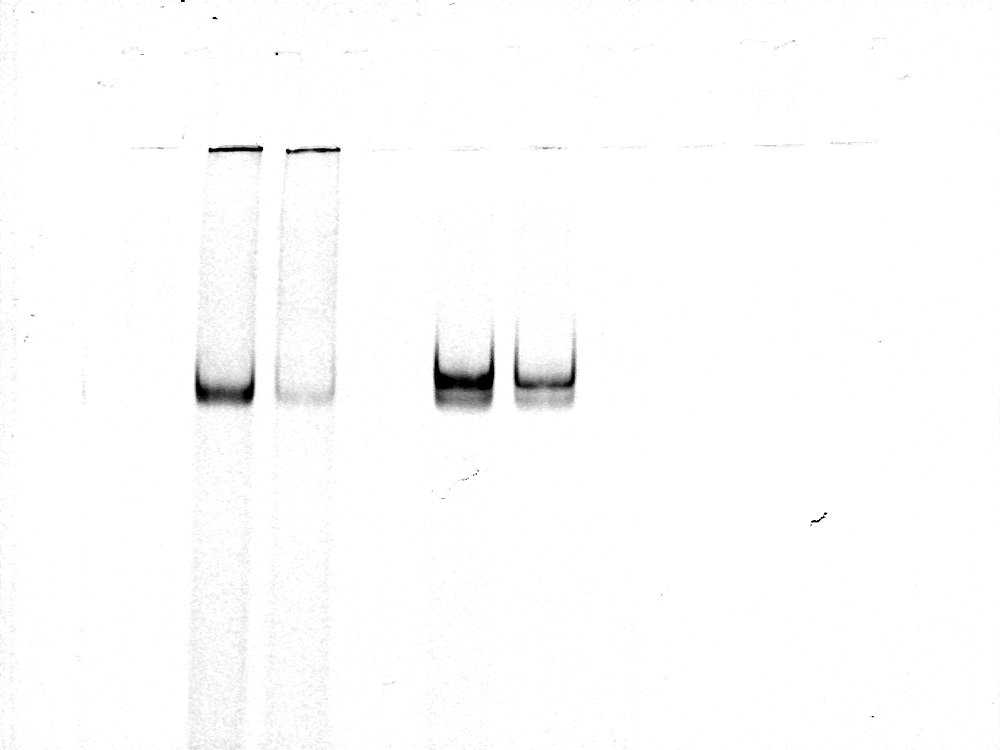

Supplement: Figure 1—source data 1. [file elife-83409-fig1-data1.zip › FIgure 1 - Source Data/RecBCD EMSA 170622-[Cy5] Figure1F.jpg]

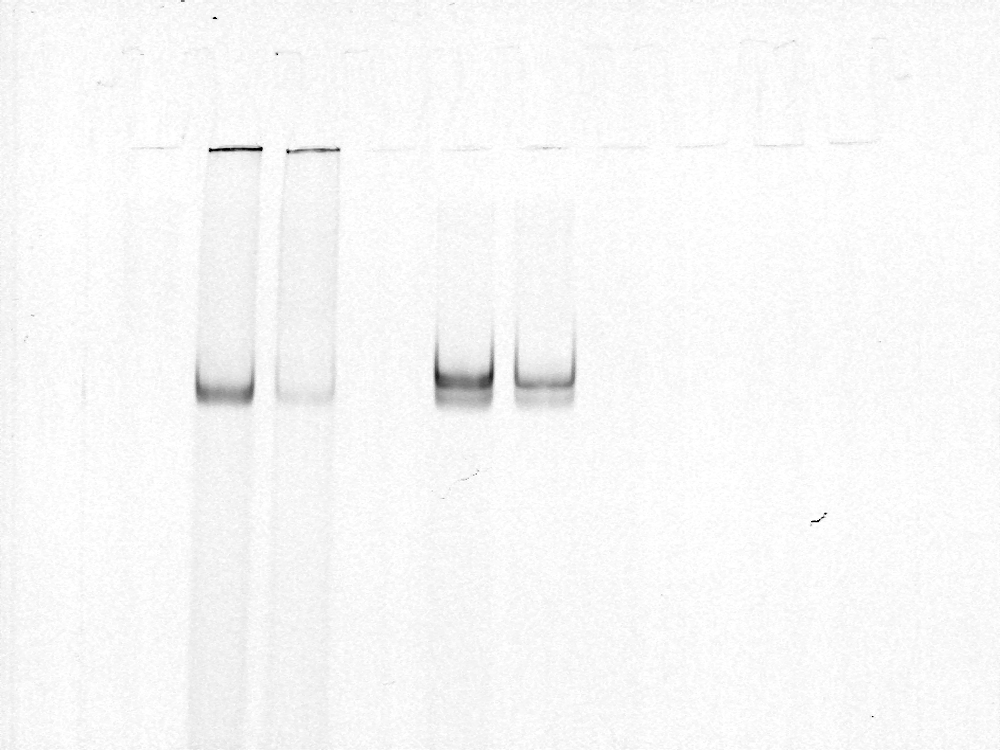

Supplement: Figure 1—source data 1. [file elife-83409-fig1-data1.zip › FIgure 1 - Source Data/RecBCD EMSA 170622-[Cy5] Figure1F.tif]
