# Supplementary material for: Structures of RecBCD in complex with phage-encoded inhibitor proteins reveal distinctive strategies for evasion of a bacterial immunity hub
Source: eLife. 2022 Dec 19;11:e83409. doi: 10.7554/eLife.83409 (PMC9836394; doi:10.7554/eLife.83409)
Supplement: Figure 3—source data 1. [file elife-83409-fig3-data1.docx]

**Figure 3 – Source Data 1**. Complete list of interactions between the RecBCD complex and gp5.9. The relevant chains, amino acids and atoms are indicated using the nomenclature from the PDB files. Note that no interactions are formed between RecD and gp5.9. Interactions were determined using ePISA (1).

| **Gp5.9 (Chain P or Q)** | **RecBCD (Chain B or C)** | **Interaction** |
| --- | --- | --- |
| P:ARG 10[ N ] | B:GLN 562[ OE1] | H-bond |
| P:ASP 11[ N ] | B:GLN 562[ OE1] | H-bond |
| P:THR 7[ O ] | B:GLN 566[ NE2] | H-bond |
| P:ASP 11[ OD1] | B:ARG 761[ NH2] | Salt bridge |
| P:ASP 11[ OD1] | B:ARG 761[ NH1] | Salt bridge |
| P:ASP 11[ OD2] | B:ARG 761[ NE ] | Salt bridge |
| P:ASP 11[ OD2] | B:ARG 761[ NH2] | Salt bridge |
| P:ASP 15[ OD2] | B:ARG 822[ NH1] | Salt bridge |
| P:GLU 36[ OE1] | B:LYS 264[ NZ ] | Salt bridge |
| P:GLU 36[ OE2] | B:LYS 264[ NZ ] | Salt bridge |
|  |  |  |
| P:TRP 13[ NE1] | C:GLY1077[ O ] | H-bond |
| P:GLN 17[ NE2] | C:ASN1078[ OD1] | H-bond |
| P:GLN 17[ NE2] | C:GLN1073[ OE1] | H-bond |
| P:ARG 3[ NH1] | C:GLU1100[ OE2] | Salt bridge |
| P:ARG 10[ NH2] | C:GLU1076[ OE1] | Salt bridge |
| P:GLU 24[ OE2] | C:LYS1070[ NZ ] | Salt bridge |
|  |  |  |
| Q:ASP 4[ OD1] | B:GLN 562[ NE2] | H-bond |
| Q:MET 35[ SD ] | B:ARG 255[ NH1] | H-bond |
| Q:ASN 49[ OD1] | B:ARG 254[ NH1] | H-bond |
| Q:ASP 4[ OD1] | B:ARG 561[ NH2] | Salt bridge |
| Q:ASP 4[ OD2] | B:ARG 561[ NH2] | Salt bridge |
| Q:ASP 4[ OD2] | B:ARG 561[ NE ] | Salt bridge |
| Q:ASP 21[ OD1] | B:ARG 824[ NH2] | Salt bridge |
| Q:GLU 24[ OE1] | B:ARG 824[ NH2] | Salt bridge |
| Q:GLU 24[ OE1] | B:ARG 824[ NE ] | Salt bridge |
| Q:GLU 24[ OE2] | B:ARG 824[ NH2] | Salt bridge |
| Q:GLU 24[ OE2] | B:ARG 824[ NE ] | Salt bridge |
| Q:ASP 38[ OD2] | B:ARG 255[ NE ] | Salt bridge |
| Q:ASP 38[ OD2] | B:ARG 255[ NH1] | Salt bridge |
| Q:GLU 39[ OE1] | B:ARG 255[ NE ] | Salt bridge |
| Q:GLU 39[ OE1] | B:ARG 255[ NH1] | Salt bridge |
| Q:GLU 39[ OE1] | B:ARG 255[ NH2] | Salt bridge |
| Q:GLU 45[ OE2] | B:ARG 254[ NH1] | Salt bridge |
| Q:GLU 45[ OE2] | B:ARG 254[ NE ] | Salt bridge |
|  |  |  |
| Q:THR 7[ OG1] | C:GLN1096[ OE1] | H-bond |
| Q:SER 22[ OG ] | C:GLN1065[ NE2] | H-bond |
| Q:ARG 3[ NE ] | C:ASP1086[ OD1] | Salt bridge |
| Q:ARG 3[ NH2] | C:ASP1086[ OD2] | Salt bridge |
| Q:ARG 3[ NH2] | C:GLU1076[ OE1] | Salt bridge |
| Q:ASP 11[ OD1] | C:ARG1068[ NH2] | Salt bridge |
| Q:ASP 11[ OD2] | C:ARG1068[ NH2] | Salt bridge |
| Q:ASP 15[ OD2] | C:ARG1068[ NE ] | Salt bridge |
| Q:ASP 15[ OD2] | C:ARG1068[ NH2] | Salt bridge |

1. Krissinel E, Henrick K. Inference of macromolecular assemblies from crystalline state. J Mol Biol. 2007;372(3):774-97.
