# Supplementary material for: Structures of RecBCD in complex with phage-encoded inhibitor proteins reveal distinctive strategies for evasion of a bacterial immunity hub
Source: eLife. 2022 Dec 19;11:e83409. doi: 10.7554/eLife.83409 (PMC9836394; doi:10.7554/eLife.83409)
Supplement: Figure 4—source data 1. [file elife-83409-fig4-data1.docx]

**Figure 4 – Source Data 1**. Complete list of interactions between the RecBCD complex and Abc2. The relevant chains, amino acids and atoms are indicated using the nomenclature from the PDB files. Note that no interactions are formed between RecD and Abc2. As discussed in the text, the structure is suggestive of interactions between RecB and Abc2 but these were not modelled due to poor electron density for the C-terminal region of Abc2. Interactions were determined using ePISA (1).

| **Abc2 (Chain X)** | **RecBCD (Chain C)** | **Interaction** |
| --- | --- | --- |
| A:ARG 13[ NH1] | C:GLU 322[ O ] | H-bond |
| A:CYS 14[ N ] | C:ASP 324[ O ] | H-bond |
| A:SER 15[ N ] | C:ASP 324[ O ] | H-bond |
| A:GLY 16[ N ] | C:PHE 326[ O ] | H-bond |
| A:TYR 30[ OH ] | C:GLU 24[ OE2] | H-bond |
| A:LYS 44[ NZ ] | C:SER 320[ O ] | H-bond |
| A:LYS 44[ NZ ] | C:SER 320[ OG ] | H-bond |
| A:LYS 44[ NZ ] | C:LEU 317[ O ] | H-bond |
| A:ARG 47[ NH2] | C:GLU 322[ OE2] | H-bond |
| A:GLY 16[ O ] | C:ASP 328[ N ] | H-bond |
| A:ILE 33[ O ] | C:GLN 321[ NE2] | H-bond |
| A:MET 34[ O ] | C:ARG 3[ NH1] | H-bond |
| A:LEU 36[ O ] | C:GLN 321[ NE2] | H-bond |
| A:GLU 39[ OE2] | C:GLU 322[ N ] | H-bond |
| A:GLU 45[ O ] | C:LYS 252[ NZ ] | H-bond |
| A:ALA 3[ N ] | C:ASP 328[ OD2] | Salt bridge |
| A:ARG 13[ NH1] | C:ASP 324[ OD1] | Salt bridge |
| A:LYS 41[ NZ ] | C:ASP 316[ OD1] | Salt bridge |
| A:ARG 47[ NH1] | C:ASP 324[ OD1] | Salt bridge |
| A:ARG 47[ NH2] | C:GLU 322[ OE2] | Salt bridge |
| A:HIS 48[ NE2] | C:GLU 322[ OE2] | Salt bridge |

1. Krissinel E, Henrick K. Inference of macromolecular assemblies from crystalline state. J Mol Biol. 2007;372(3):774-97.
